# Involvement of the liver-gut peripheral neural axis in nonalcoholic fatty liver disease pathologies via hepatic HTR2A

**DOI:** 10.1242/dmm.049612

**Published:** 2022-07-26

**Authors:** Takashi Owaki, Kenya Kamimura, Masayoshi Ko, Itsuo Nagayama, Takuro Nagoya, Osamu Shibata, Chiyumi Oda, Shinichi Morita, Atsushi Kimura, Takeki Sato, Toru Setsu, Akira Sakamaki, Hiroteru Kamimura, Takeshi Yokoo, Shuji Terai

**Affiliations:** 1 Division of Gastroenterology and Hepatology, Graduate School of Medical and Dental Sciences, Niigata University, Niigata 951-8510, Japan; 2 Department of General Medicine, Niigata University School of Medicine, Niigata 951-8510, Japan

**Keywords:** Fatty liver, Autonomic neuron, 5-HT, Antagonist, Brain

## Abstract

Serotonin (5-HT) is one of the key bioamines of nonalcoholic fatty liver disease (NAFLD). Its mechanism of action in autonomic neural signal pathways remains unexplained; hence, we evaluated the involvement of 5-HT and related signaling pathways via autonomic nerves in NAFLD. Diet-induced NAFLD animal models were developed using wild-type and melanocortin 4 receptor (MC4R) knockout (MC4RKO) mice, and the effects of the autonomic neural axis on NAFLD physiology, 5-HT and its receptors (HTRs), and lipid metabolism-related genes were assessed by applying hepatic nerve blockade. Hepatic neural blockade retarded the progression of NAFLD by reducing 5-HT in the small intestine, hepatic HTR2A and hepatic lipogenic gene expression, and treatment with an HTR2A antagonist reproduced these effects. The effects were milder in MC4RKO mice, and brain 5-HT and HTR2C expression did not correlate with peripheral neural blockade. Our study demonstrates that the autonomic liver-gut neural axis is involved in the etiology of diet-induced NAFLD and that 5-HT and HTR2A are key factors, implying that the modulation of the axis and use of HTR2A antagonists are potentially novel therapeutic strategies for NAFLD treatment.

This article has an associated First Person interview with the first author of the paper.

## Abstract (precis)

**Summary:** The hepatic-gut neural axis plays a role in NAFLD progression via serotonin and the serotonin receptor HTR2A in hepatocytes, suggesting that HTR2A antagonists are potential therapeutic agents for NAFLD.

## INTRODUCTION

Autonomic nerve pathways maintain biological homeostasis and are implicated in various pathologies, including nonalcoholic fatty liver disease (NAFLD) (Teratani et al., 2020; Kamiya et al., 2019; Hayakawa and Wang, 2017; Imai, 2018; Imai et al., 2008). The prevalence of NAFLD is increasing (Younossi et al., 2016), and its diverse and complicated etiology, ranging from obesity and dyslipidemia to abnormal hormone secretion, genetic factors and the gut-liver axis (Tilg and Moschen, 2010; Tilg et al., 2020; Ko et al., 2021; Wiest et al., 2017), has rendered defining a standard therapeutic method rather challenging.

Overconsumption of palatable energy-dense foods is associated with obesity and metabolic disorders that lead to NAFLD. The physiology involves the central neural network in the brain and the peripheral autonomic neural pathway, which connect various organs. The central network consists of the melanocortin pathway, which includes pro-opiomelanocortin (POMC), melanocyte-stimulating hormones and the melanocortin 4 receptor (MC4R), and the agouti-related protein (AgRP) and neuropeptide Y (NPY) pathway in the brain, which are mostly independent from peripheral humoral factors due to the blood-brain barrier (de Andrade Silva et al., 2021; Vella et al., 2011; Arens et al., 2003). Although the role of the neural network in the brain in appetite control has been examined (Valdivia et al., 2014; Baldini and Phelan, 2019), little is known about the involvement of the peripheral autonomic neural pathway in NAFLD etiology (Amir et al., 2020; Hurr et al., 2019; Houghton et al., 2019; Nishio et al., 2017; Mizuno and Ueno, 2017; Sabath et al., 2015; Oben et al., 2004; Borovikova et al., 2000) and its relationship to the central neural pathway.

We have previously reported that the peripheral autonomic neural pathway contributes to liver regeneration by promoting serotonin (5-hydroxytryptamine; 5-HT) synthesis in the small intestine (SI) through the afferent sympathetic nerves and the brain (Inoue et al., 2018; Kamimura et al., 2018). Additionally, we have demonstrated that gastric ghrelin (GHRL) activation relays the afferent neural signal from the stomach to the hypothalamus to release hepatic insulin-like growth factor-1 (IGF-1), which then reverses fatty changes (Nagoya et al., 2020). Recently, we have also shown that modulation of 5-HT synthesis in the SI through the gut-liver neural axis ameliorates fatty and fibrotic changes in NAFLD and that it involves tight junction protein expression, microbiota diversity and short chain fatty acids (Ko et al., 2021). Thus, autonomous nerve signals appear to be involved in the pathogenesis and progression of NAFLD through the inter-organ neural axis.

5-HT is a multifunctional bioamine with important signaling roles in a range of physiological pathways, including smooth muscle contraction, platelet aggregation, appetite, anxiety (Kroeze et al., 2002; Berger et al., 2009), energy metabolism in peripheral tissues (Choi et al., 2020; Martin et al., 2020; Namkung et al., 2015, 2018) and liver biology (Ruddell et al., 2008). It acts as a neurotransmitter in the brain; however, it is predominantly (90%) secreted by the enterochromaffin cells of the gastrointestinal tract, known as peripheral 5-HT (Martin et al., 2020, 2017), and executes diverse functions through tissue-specific receptors (Berger et al., 2009). No fewer than 15 receptors have been reported (Kroeze et al., 2002; Ruddell et al., 2008), and those in the liver contribute to regeneration (Inoue et al., 2018; Lesurtel et al., 2006; Ebrahimkhani et al., 2011) and steatosis (Namkung et al., 2018; Haub et al., 2011; Zhang et al., 2020; Crane et al., 2015; Li et al., 2018; Choi et al., 2018).

As gut-specific tryptophan hydroxylase 1 (*Tph1*) knockout mice and liver-specific *Htr2a* knockout mice are resistant to high-fat diet (HFD)-induced hepatic steatosis, the gut TPH1-liver-HTR2A axis is a promising drug target for NAFLD (Crane et al., 2015; Choi et al., 2018). However, the mechanisms regulating 5-HT and 5-HT receptor (HTR) expression and their relationship with the central neural pathway are not well characterized. For this purpose, in this study, we used melanocortin 4 receptor (MC4R) knockout (KO) mice as a model to test the central melanocortin pathway in NAFLD, as MC4R expression is limited in the brain, and 5-HT and its receptor HTR2C in the brain inhibit appetites via the MC4R signaling pathway (Lam et al., 2010). MC4R mutations are associated with early-onset obesity and NAFLD in humans, and MC4RKO mice show the nonalcoholic steatohepatitis (NASH)-like hepatic phenotype by exhibiting an increased appetite when fed with a HFD (Vella et al., 2011; Arens et al., 2003; Itoh et al., 2011; Miyamura et al., 2011). In addition, we have proven that MC4RKO mice show further activation of NPY/AgRP-producing neurons in the arcuate nucleus, which influences the appetite, than what is seen in wild-type (WT) mice, as well as a higher expression of gastric ghrelin and IGF-1, which slows down the progression of steatoheptitis (Nagoya et al., 2020). Then, we analyzed the expression of 5-HT, HTRs and lipid metabolism-related genes using diet-induced and central neuron-dependent murine NAFLD models of MC4RKO mice (Nagoya et al., 2020; Itoh et al., 2011) with or without neural signal blockade of the liver-gut autonomic neural axis. Moreover, the effects of central 5-HT and its receptor were examined to ascertain their relationship with peripheral 5-HT and the feasibility of serum 5-HT concentration as a biomarker was explored in both mouse models and samples from NAFLD patients.

## RESULTS

### Effect of autonomic nerve signal on food intake, body weight and liver weight in NAFLD mouse models

**Fig. 1.**
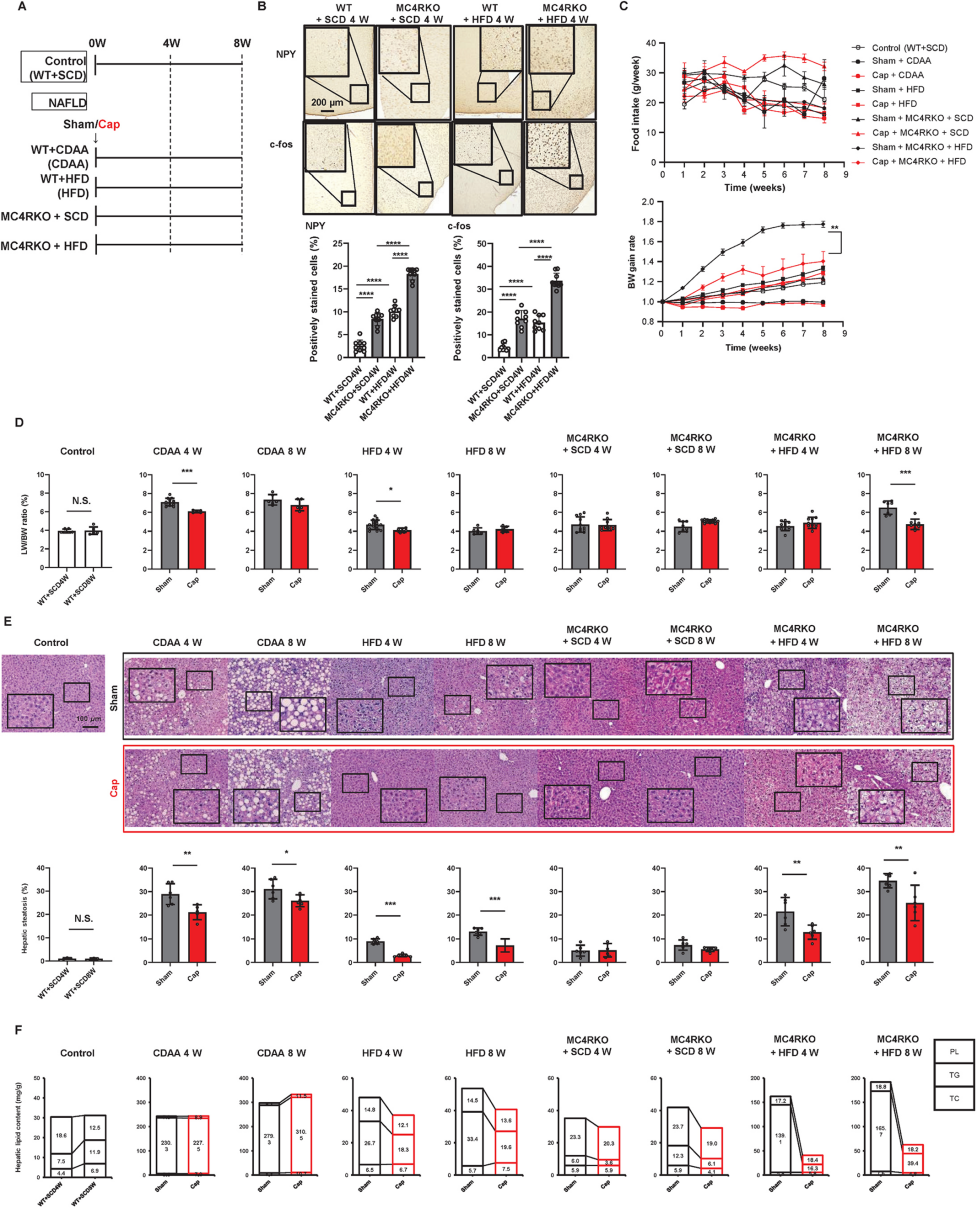
**Effect of autonomic nerve signal transduction on physiological conditions, hepatic steatosis and lipid content in NAFLD and PH mouse models.** (A) Experimental design. The mice were divided into control and NAFLD groups (*n*=6-8 mice per group). The control group consisted of WT mice fed SCD (WT+SCD) for 8 weeks. The NAFLD group was further classified into 16 groups; each group consisted of mice that had undergone either Sham or Cap treatment and were WT+CDAA (CDAA), WT+HFD (HFD), MC4RKO+SCD or MC4RKO+HFD for 4 or 8 weeks (*n*=6-8 mice per group). (B) NPY and c-fos expression in the hypothalamus of WT+SCD, MC4RKO+SCD, WT+HFD4W and MC4RKO+HFD4W mice. Scale bar: 200 μm. Five different sections from each of the 6-8 mice in all groups were quantitatively analyzed for fatty infiltration using ImageJ. One-way ANOVA with post-hoc Tukey's test. Each symbol represents data from one mouse. (C) Time-dependent changes in food intake and BW. The significance of differences was evaluated by two-way, repeated-measure ANOVA, followed by Tukey's multiple comparison test. (D) LW/BW ratio (*n*=6-8 mice per group). Two-tailed unpaired Welch's *t*-test. Each symbol represents data from one mouse. (E) Representative images and quantitative analysis of hepatic H&E staining. Scale bar: 100 μm. Five different sections from each of the 6-8 mice in all groups were quantitatively analyzed for fatty infiltration using ImageJ. Two-tailed unpaired Welch's *t*-test. Each symbol represents data from one mouse. (F) Hepatic lipid content (mg/g), such as phospholipid (PL), triglyceride (TG) and total cholesterol (TC), (*n*=2 for each group). Values represent the mean±s.d. (B-E) or the mean (F). N.S., not significant; *P<0.05; **P<0.01; ***P<0.001; ****P<0.0001.

Animal models were developed as described in the Materials and Methods (Fig. 1A). How the central neural network was affected by appetite between WT and MC4RKO mice was examined based on the expression of NPY and c-fos (encoded by *Fos*) in the hypothalamus (Fig. 1B). HFD increased NPY and c-fos expression as previously reported in WT mice (Valdivia et al., 2014; Baldini and Phelan, 2019) and MC4RKO mice showed the same pattern. MC4RKO mice showed higher expression of these proteins in the hypothalamus than WT mice in both standard chow diet (SCD)- and HFD-fed conditions, indicating that MC4R modification activates the central neural network regardless of the palatable energy-dense HFD administration. Calcitonin gene-related peptide staining showed that capsaicin-induced nerve blockade was most effective by 4 weeks (4W) with subsequent slow recovery (Ko et al., 2021).

To examine the effect of visceral nerve blockade on food intake, body weight (BW) and liver weight (LW) in NAFLD mice, time-dependent changes in food intake, BW gain rate (Fig. 1C) and LW/BW ratio (Fig. 1D) were calculated. There were no significant changes in food intake in any of the NAFLD mice, with or without visceral nerve blockage. The MC4RKO+HFD mice showed significant BW gain compared to control mice (WT+SCD); in contrast, visceral nerve blockade by capsaicin (Cap) treatment led to significant inhibition of BW gain (Sham+MC4RKO+HFD versus Cap+MC4RKO+HFD; *P<*0.01; Fig. 1C). HFD-fed mice showed greater BW gain than SCD-fed control mice. LW/BW ratio showed a significant increase in choline-deficient defined L-amino-acid (CDAA)-fed, HFD-fed and MC4RKO mice compared to control mice (WT+SCD4W versus Sham+CDAA4W, WT+SCD8W versus Sham+CDAA8W, WT+SCD4W versus Sham+HFD4W, WT+SCD4W versus Sham+MC4RKO+HFD4W and WT+SCD8W versus Sham+MC4RKO+HFD8W; *P<*0.05) and was significantly lower with visceral nerve blockade in the CDAA 4W, HFD 4W and MC4RKO+HFD 8W groups (Fig. 1D). Altogether, these results suggest that neural signaling from the liver through the visceral nerve contributes to LW and BW gain in HFD-induced NAFLD models, despite no difference in food intake.

### Effect of autonomic nerve signal on hepatic steatosis in NAFLD mice

To determine whether signal transduction through the autonomic nerve system contributes to hepatic steatosis progression, we histologically assessed the amount of hepatic fatty tissue and measured hepatic lipid content in NAFLD mouse models, with or without visceral nerve blockade, and used control mice for comparison. Hepatic steatosis was defined as areas of fatty tissue, as reported previously (Vrekoussis et al., 2009) (Fig. 1E). Hepatic steatosis was significantly greater in CDAA-fed WT, HFD-fed WT, SCD-fed MC4RKO and HFD-fed MC4RKO mice compared to control mice (*P<*0.05). Although MC4RKO mice fed with SCD showed no significant change after nerve blockade, CDAA-fed WT, HFD-fed WT and HFD-fed MC4RKO mice showed inhibition of hepatic fatty infiltration after visceral nerve blockade for 8 weeks. Analysis of hepatic lipid content in HFD-fed WT and MC4RKO mice showed a remarkable increase in triglycerides (TG) with no significant changes in total cholesterol (TC) or phospholipids (PL), which was recovered by the visceral neural blockade (Fig. 1F). However, the recovery of hepatic steatosis and hepatic lipid content was milder in HFD-fed MC4RKO than HFD-fed WT mice. These results suggest that the peripheral visceral nerve contributes to hepatic adipose tissue accumulation in diet-induced NAFLD models by altering TG metabolism; however, the effect is independent from the central neural pathway.

### Effect of autonomic nerve signals on 5-HT expression in the SI

**Fig. 2.**
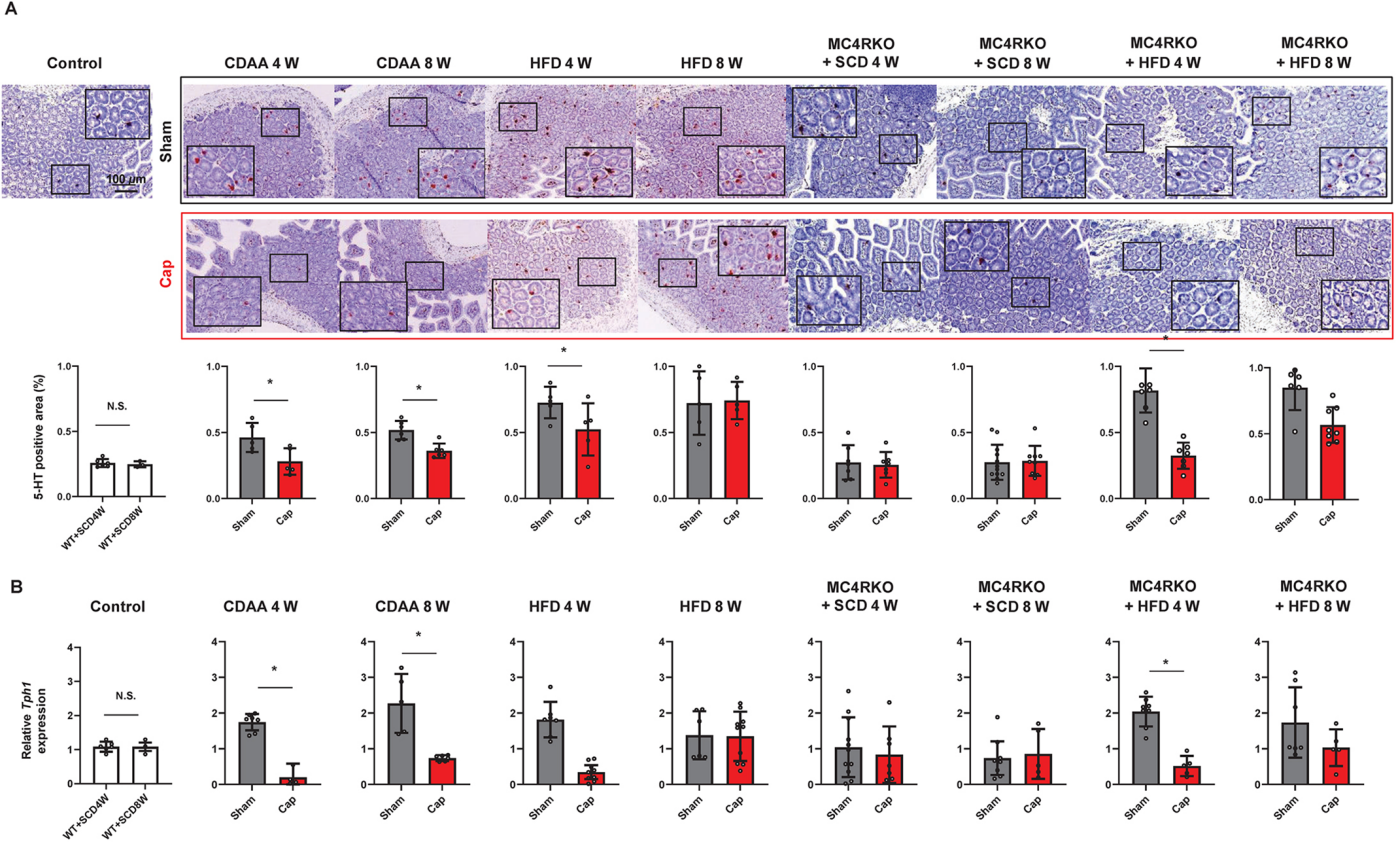
**Expression of 5-HT in the small intestine in mouse models.** (A) Representative images and quantitative analysis of 5-HT expression in the small intestine of mice. Scale bar: 100 μm. Five different sections from each of the 6-8 mice in all groups were quantitatively analyzed for 5-HT expression using ImageJ. (B) Relative mRNA expression of *Tph1* in the small intestine. *Gapdh* was used as an internal control. All values represent the mean±s.d. (*n*=6-8 mice per group); two-tailed unpaired Welch's *t*-test. Each symbol represents data from one mouse. N.S., not significant; *P<0.05.

It is known that the expression of 5-HT, a gastrointestinal hormone produced mainly in the SI, is significantly related to hepatic regeneration after injury (Lesurtel et al., 2006; Ebrahimkhani et al., 2011) and progression of fatty liver disease (Li et al., 2018; Choi et al., 2018; Wang et al., 2020), but it is inhibited by autonomic nerve signal blockade, especially that of afferent sympathetic fibers from the injured liver (Inoue et al., 2018). Therefore, we assessed 5-HT expression in the SI of NAFLD mice. Compared with controls, in all mouse models except MC4RKO+SCD mice, 5-HT expression in the SI was significantly higher (*P<*0.05). Visceral nerve blockade contributed to significant 5-HT suppression in the SI in CDAA- and HFD-fed mice, especially within 4 weeks after the neural blockade (Fig. 2A) as the effect of capsaicin slowly decreased after 4 weeks (Ko et al., 2021). The mRNA expression of *Tph1* in the SI, which encodes an enzyme catalyzing 5-HT synthesis, also showed a similar pattern, indicating visceral nerve involvement for the diet-induced NAFLD (Fig. 2B). These changes showed no difference between WT and MC4RKO mice when fed with HFD. These results imply that diet-induced NAFLD might be related to 5-HT activation in the SI regardless of central neural pathway alteration (MC4RKO). Moreover, altering 5-HT expression through visceral nerve blockade may help inhibit hepatic steatosis.

### Effects of autonomic nerve signals on the expression of hepatic *Htr2a* and *Htr2b*

**Fig. 3.**
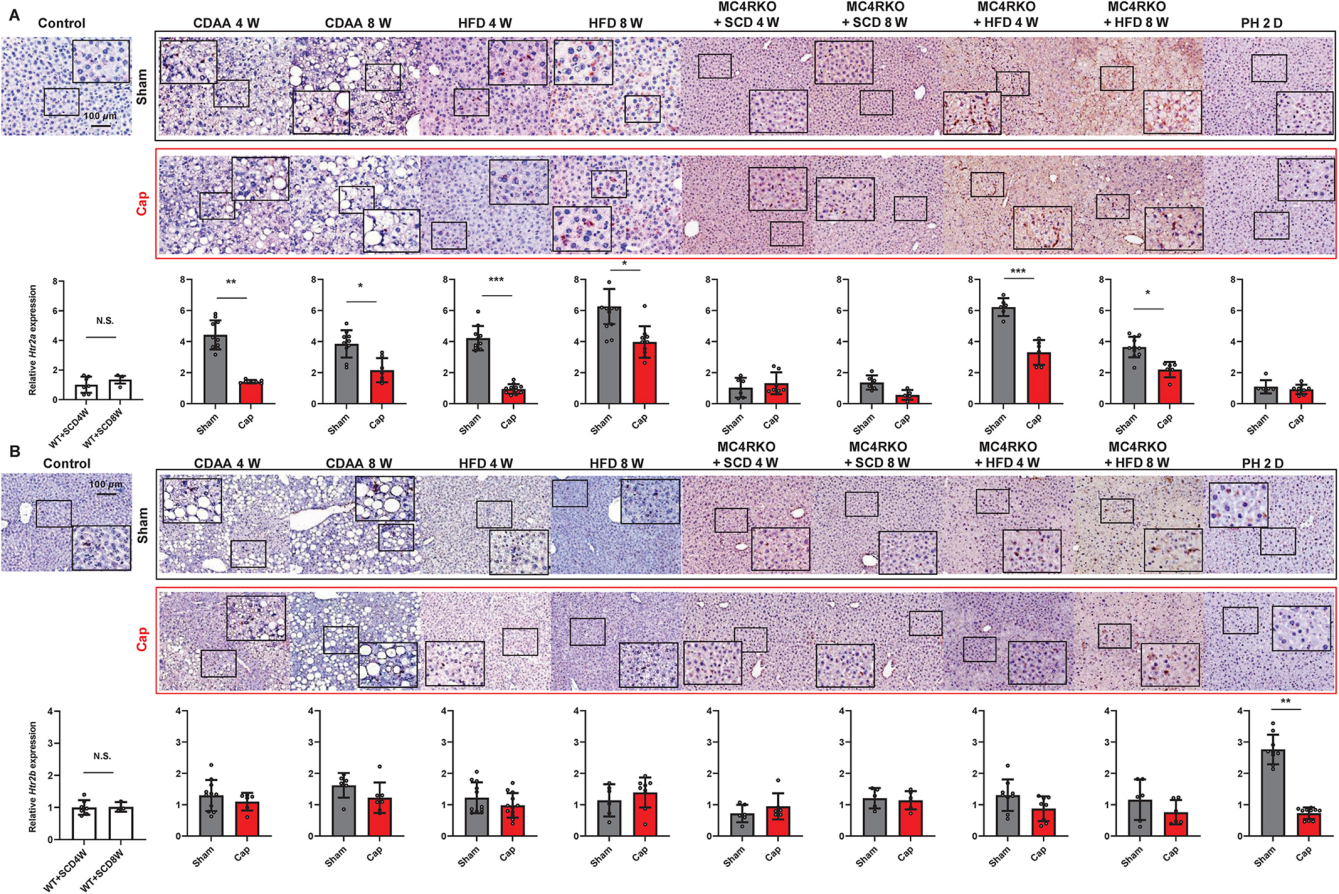
**Expression of 5-HT receptors.** (A) Hepatic expression of HTR2A in mice. Representative images of HTR2A expression in the liver of the model mice. The PH group consisted of mice that had undergone Sham or Cap treatment, followed by 70% partial hepatectomy. Scale bar: 100 μm. Panels below the images show relative mRNA expression of *Htr2a* in the liver. (B) Expression of HTR2B in the liver of NAFLD and PH mouse models. Representative images of HTR2B expression in the liver of mice. Scale bar: 100 μm. Panels below the images show relative mRNA expression of *Htr2b* in the liver. *Gapdh* was used as an internal control. All values represent the mean±s.d. (*n*=6-8 mice per group). Two-tailed unpaired Welch's *t*-test. Each symbol represents data from one mouse. N.S., not significant; *P<0.05; **P<0.01; ***P<0.001.

Among the various subtypes of HTRs expressed by hepatocytes (Li et al., 2018), HTR2A and HTR2B show a strong relationship to liver diseases as they transduce the 5-HT signal from the SI via the portal vein (Lesurtel et al., 2006; Ebrahimkhani et al., 2011; Li et al., 2018; Choi et al., 2018; Lam et al., 2010; Wang et al., 2020). Therefore, we analyzed hepatic expression of *Htr2a* and *Htr2b* by immunostaining and quantitative real-time PCR in these mouse models to determine whether the expression of these receptors was associated with modification of 5-HT expression in NAFLD mice (Fig. 3). *Htr2a* expression was significantly higher compared to controls in all NAFLD mice, except in MC4RKO+SCD mice and mice that underwent partial hepatectomy (PH) (*P<*0.05). Moreover, the visceral nerve blockade reduced *Htr2a* levels in CDAA-fed WT, HFD-fed WT and MC4RKO+HFD mice (Fig. 3A). No difference was observed between WT and MC4RKO mice when fed with HFD. In PH models, *Htr2a* expression showed no change; however, significantly higher *Htr2b* expression was observed in PH models compared to control and other models, as previously reported (Li et al., 2018), and *Htr2b* was significantly suppressed with visceral nerve blockade (Fig. 3B). These results suggest that *Htr2a* expression is related to specific dietary stress and is independent of central neural pathway alteration (MC4RKO), and that *Htr2b* expression is not associated with diet-induced NAFLD. In addition, visceral nerve signaling is involved in HTR expression in the liver.

### Effect of autonomic nerve signaling on lipid metabolism-related gene expression

**Fig. 4.**
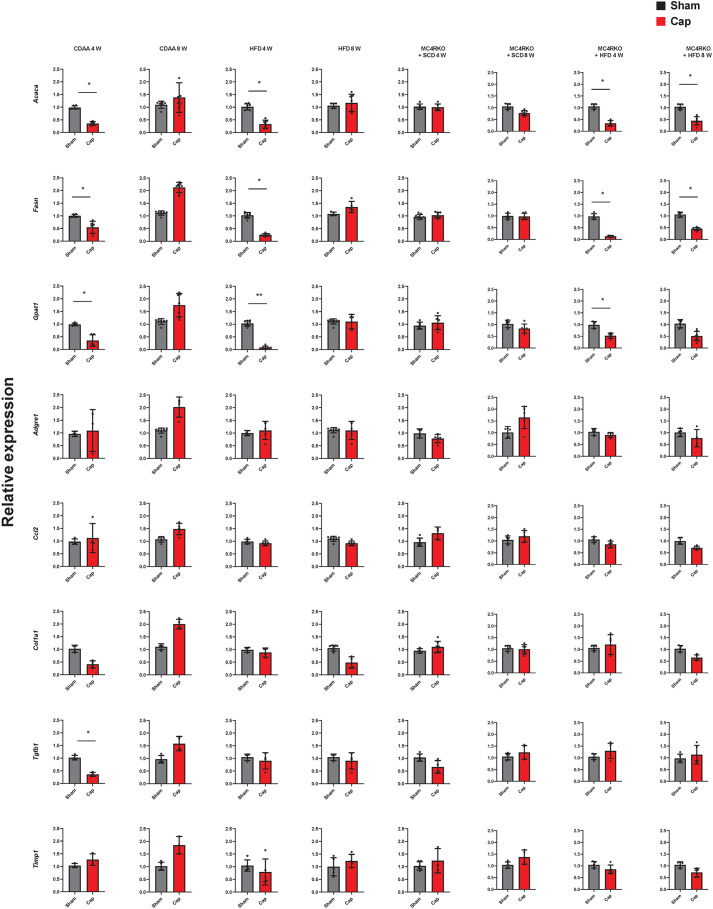
**Analysis of the effect of Cap treatment on lipid metabolism-, inflammation- and fibrosis-related gene expression in the liver of mice.** Relative mRNA expression of acetyl-CoA carboxylase (*Acaca*), fatty acid synthase (*Fasn*), glycerol-3-phosphate acyltransferase (*Gpat1*), adhesion G protein-coupled receptor E1 (*Adgre1*), C-C motif chemokine ligand 2 (*Ccl2*), collagen type I alpha 1 (*Col1a1*), transforming growth factor-β1 (*Tgfb1*) and tissue inhibitor of metalloproteinases 1 (*Timp1*) in the liver are shown. *Gapdh* was used as an internal control. Values represent the mean±s.d. (*n*=6-8 mice per group). *P<0.05; **P<0.01; two-tailed unpaired Welch's *t*-test. Each symbol represents data from one mouse.

To probe mechanisms underlying hepatic steatosis suppression through the 5-HT-HTR2A axis and the autonomic nervous system, we evaluated the expression of various lipid metabolism-related genes. Specifically, in CDAA, HFD and MC4RKO+HFD models, the expression of genes involved in fatty acid synthesis, such as *Acaca*, *Fasn* and *Gpat1* (also known as *Gpam*), remained suppressed up to 4 weeks after visceral nerve blockade, but recovered by 8 weeks (Fig. 4), which is consistent with changes in hepatic steatosis, 5-HT and *Tph1* expression (Figs 1 and 2), due to the recovery from capsaicin-induced nerve blockade (Ko et al., 2021).

No effects on the expression of these genes were observed in MC4RKO-fed SCD (MC4RKO+SCD) mice. Additionally, no significant changes were observed in the expression of genes involved in fatty acid oxidation (*Cpt1a*), very low-density lipoprotein (VLDL) secretion (*Mttp*), lipogenic transcription factors (*Pparg* and *Srebf1*) (data not shown), inflammation (*Adgre1* and *Ccl2*) or fibrosis (*Col1a1*, *Tgfb1* and *Timp1*) (Fig. 4), suggesting that suppression of 5-HT and *Htr2a* expression by visceral nerve blockade inhibits fatty acid synthesis in the liver of diet-induced NAFLD mice.

### HTR2A antagonist inhibits hepatic steatosis in mice

**Fig. 5.**
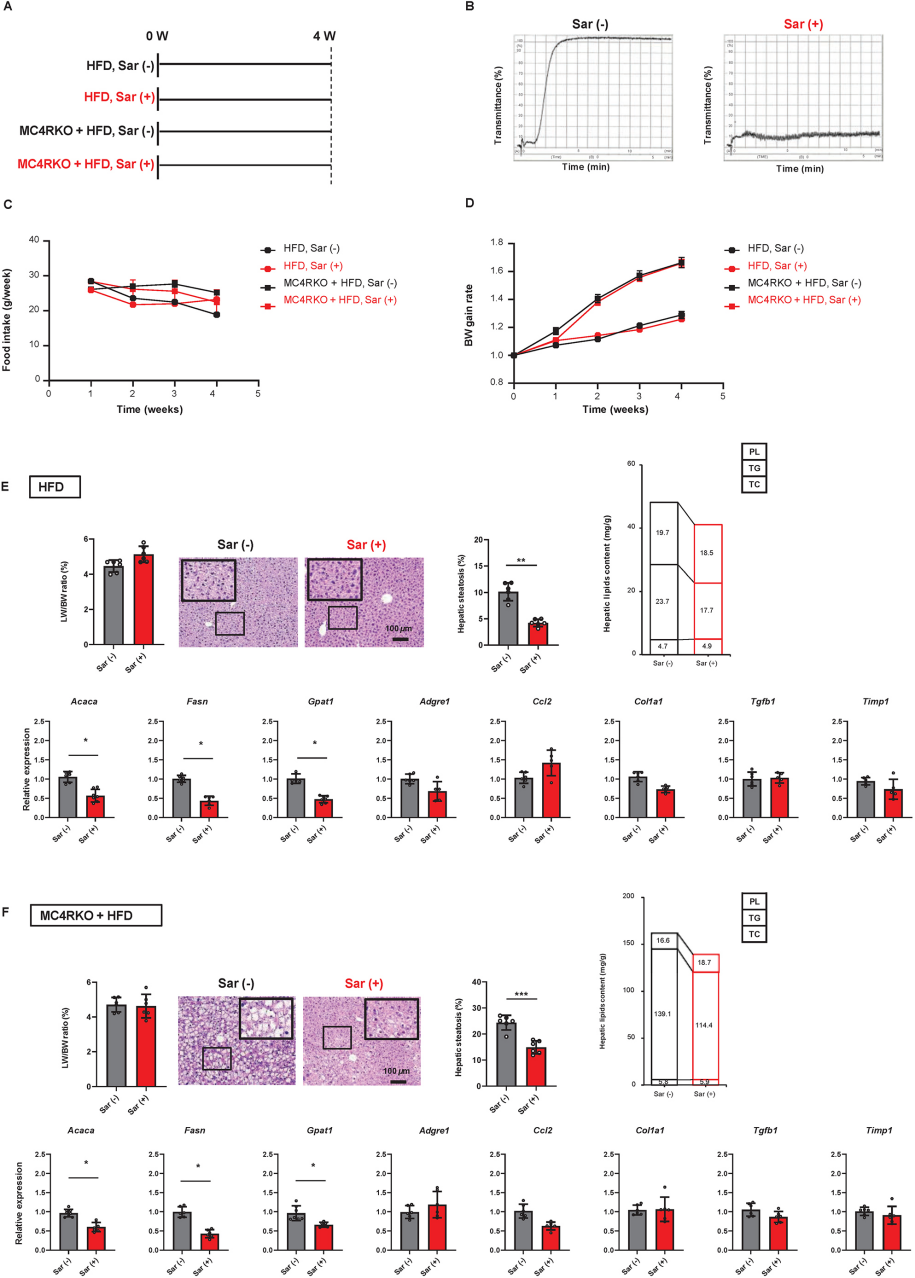
**Effect of HTR2A antagonist on mice.** (A) Experimental design. Mice were divided into four groups (*n*=5 mice for each group). Sarpogrelate, Sar. (B) Changes in platelet aggregation with and without Sar. (C) Time-dependent change in food intake. (D) Time-dependent change in BW. (E,F) Results from a group of control mice (E) or MC4RKO mice (F) fed HFD for 4 weeks, with and without Sar (*n*=5 mice for each group). Both groups show the following results: LW/BW ratio, representative images and quantitative analysis of H&E staining of the liver, hepatic lipid content (mg/g) such as PL, TG and TC, and relative mRNA expression of *Acaca*, *Fasn*, *Gpat1*, *Adgre1*, *Ccl2*, *Col1a1*, *Tgfb1* and *Timp1* in the liver. *Gapdh* was used as an internal control. Each symbol represents data from one mouse. Scale bars: 100 μm. Five different sections from each animal in all groups were quantitatively analyzed for fatty infiltration using ImageJ. Values represent the mean±s.d. or the mean (*n*=5 mice per group). *P<0.05; two-tailed unpaired Welch's *t*-test.

To further confirm the effect of the 5-HT-liver-HTR2A axis on diet-indued hepatic steatosis through modification of hepatic lipogenesis genes, the HTR2A antagonist sarpogrelate was administered to HFD-fed WT and HFD-fed MC4RKO mice. The experimental design is shown in Fig. 5A. First, sarpogrelate efficacy was confirmed by ascertaining the suppression of platelet aggregation (Fig. 5B). Sarpogrelate did not alter food intake (Fig. 5C), BW gain (Fig. 5D) or LW/BW ratio in both animal models (Fig. 5E,F). Importantly, hepatic steatosis and TG content were significantly lower in the sarpogrelate group, and the expression of genes related to fatty acid synthesis (*Acaca*, *Fasn* and *Gpat1*) was also reduced. Importantly, the reduction of these parameters in HFD-fed MC4RKO mice was milder than in HFD-fed WT mice, similar to the results shown in Fig. 1E,F. These results suggest that HTR2A-signal blockade in the liver suppresses the diet-induced expression of fatty acid synthesis-related genes and hepatic steatosis.

### Serum biochemical analysis

Although the CDAA and HFD models showed higher serum levels of aspartate aminotransaminase (AST), alanine aminotransferase (ALT) and alkaline phosphatase (ALP) than controls, no significant changes were seen upon visceral nerve blockade or HTR2A antagonist administration (Table S4). However, HFD-fed WT and HFD-fed MC4RKO mice exhibited a tendency of reduced TC and TG levels upon visceral nerve blockade (Table S4).

### Serum 5-HT levels in patients with NAFLD

**Fig. 6.**
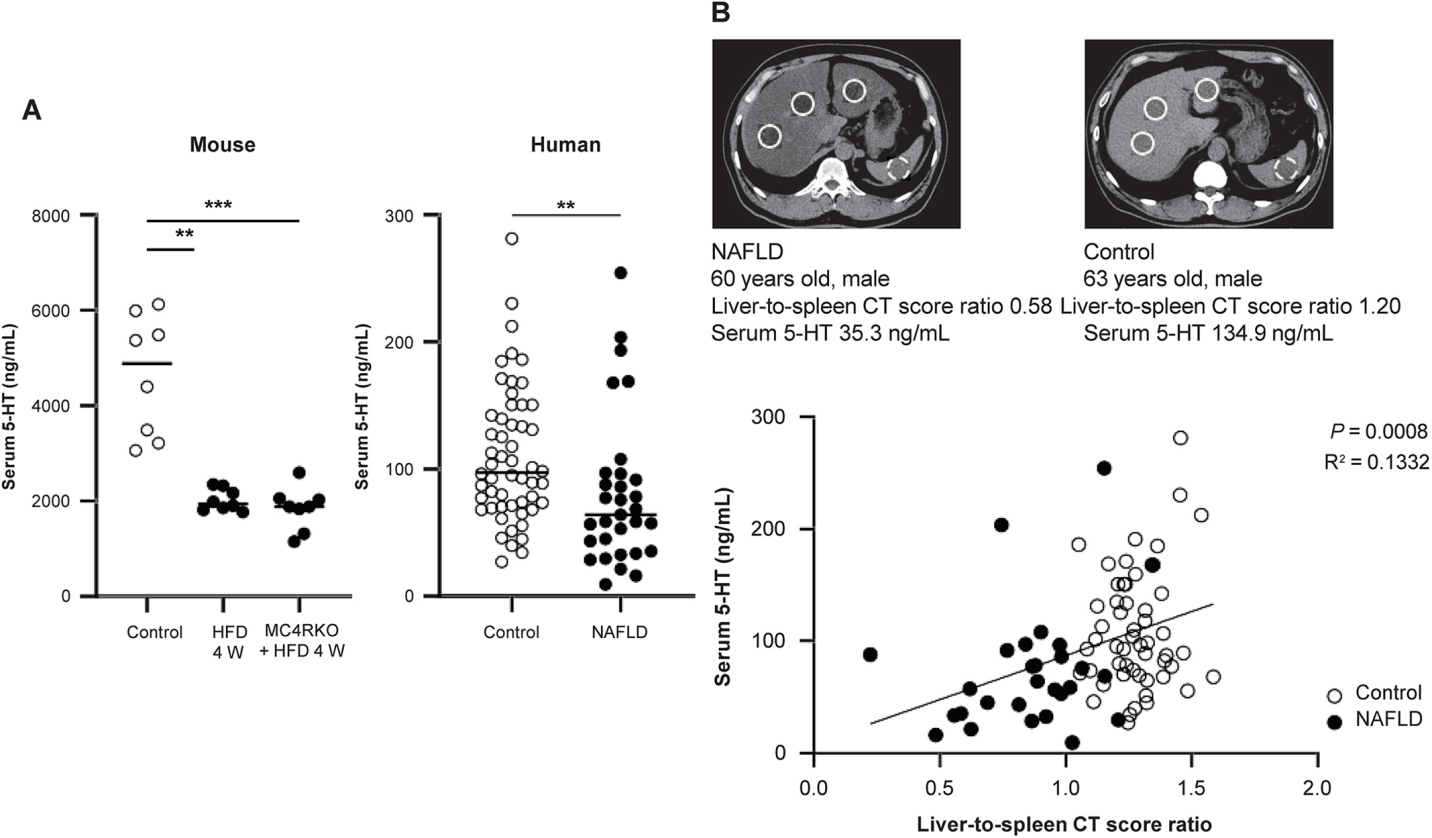
**Relationship between 5-HT and NAFLD.** (A) Serum 5-HT levels in NAFLD mouse models and patients with NAFLD. Values represent the median. **P<0.01; ***P<0.001; Kruskal–Wallis test with post-hoc Dunn's test (mouse, *n*=8 mice per group) or Mann–Whitney *U* test (human; *n*=54, control and *n*=31, NAFLD group). Each symbol represents data from one mouse or one patient. (B) Representative CT images from the NAFLD and control groups. White circles indicate the regions of interest for the CT score in the liver. Correlations were evaluated by performing Pearson's correlation test (*n*=54, control and *n*=31, NAFLD group). Each symbol represents data from one patient.

As 5-HT produced in the SI is transported via portal circulation to the liver for subsequent binding with HTR2A, we examined the kinetics of serum 5-HT levels in HFD-fed WT and HFD-fed MC4RKO mice, and in patients with fatty liver disease, to determine whether 5-HT levels reflect hepatic steatosis. 5-HT levels in NAFLD mice (Fig. 6A) and in patients with histologically confirmed NAFLD (Fig. 6B) were significantly lower compared to the control groups. Further, clinical data revealed that more severe fatty infiltration in the liver, reflected by lower liver-to-spleen computed tomography (CT) scores, was related to lower serum 5-HT levels, with a significant positive correlation between the serum 5-HT and liver-to-spleen CT score ratio (Fig. 6B). These results suggest that 5-HT released from the SI might bind to hepatic HTR2A, therefore reducing its serum concentration and activating hepatic lipid synthesis.

### Expression of 5-HT and HTR2C in the brain of NAFLD mice

**Fig. 7.**
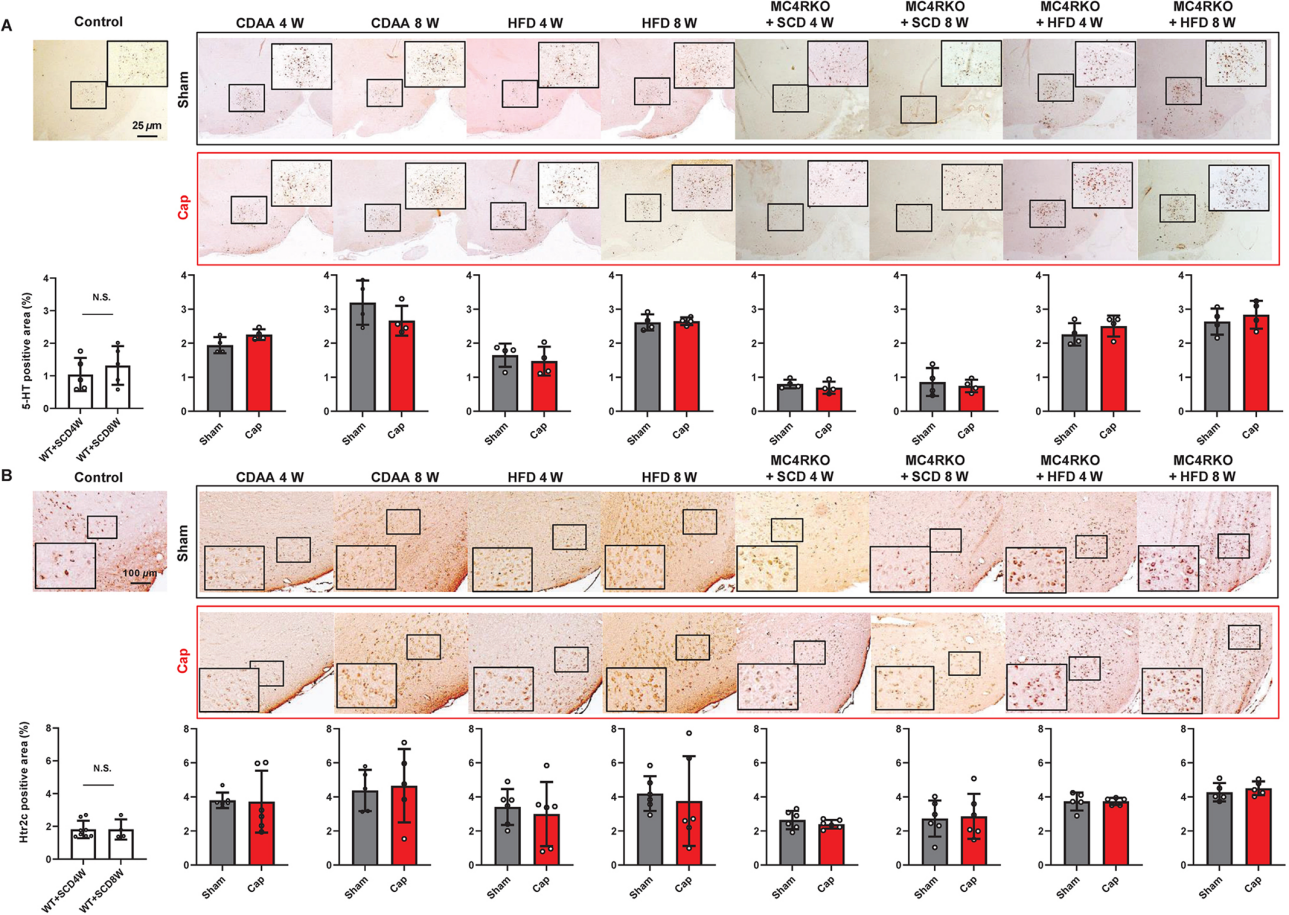
**Expression of 5-HT and HTR2C in the brain of mouse models.** (A) Representative images and quantitative analysis of 5-HT expression in the brain of mice. Scale bar: 25 μm. Five different sections from each of the 6-8 mice in all groups were analyzed for 5-HT expression using ImageJ. (B) Representative images and quantitative analysis of HTR2C expression in the brain of mice. Scale bar: 100 μm. Five different sections from each of the 6-8 mice in all groups were analyzed for HTR2C expression using ImageJ. Values represent the mean±s.d. (*n*=6-8 mice per group). N.S., not significant; two-tailed unpaired Welch's *t*-test. Each symbol represents data from one mouse.

Given that 5-HT in the brain has been reported to suppress appetite by acting on HTR2C in the hypothalamus via the MC4R signaling pathway and that peripheral 5-HT does not cross the blood brain barrier (Lam et al., 2010), the expression of 5-HT and HTR2C in the hypothalamus, with or without autonomic nerve blockade, was analyzed to examine whether 5-HT in the brain contributes to hepatic steatosis development in the mouse models. Compared to controls, the expression of 5-HT and HTR2C in the hypothalamus was significantly higher in NAFLD mice, especially in the CDAA-fed WT, HFD-fed WT and HFD-fed MC4RKO models (Fig. 7A,B). However, unlike 5-HT in the SI and HTRs in the liver, visceral nerve blockade did not alter the expression of 5-HT or HTR2C in the hypothalamus (Fig. 7). These results suggest that fat loading increases the expression of central 5-HT, which then inhibits feeding, independently of nerve signals from the liver.

## DISCUSSION

We demonstrate that autonomic nerve signals from livers exposed to CDAA and HFD-induced fatty changes activate 5-HT secretion into the serum from the SI, which then acts on HTR2A on the hepatocytes that is involved in steatosis in NAFLD. Specifically, this 5-HT-liver-HTR2A axis contributes to the activation of steatosis-related gene expression, which modifies TG metabolism, BW and LW gain, and hepatic steatosis in CDAA-fed WT, HFD-fed WT and even HFD-fed MC4RKO mice. These results are supported by the effects of 5-HT secretion as seen in CDAA- and HFD-fed mice (Amir et al., 2020; Hurr et al., 2019; Houghton et al., 2019; Nishio et al., 2017; Mizuno and Ueno, 2017; Sabath et al., 2015; Oben et al., 2004; Borovikova et al., 2000) and on HTR2A (Choi et al., 2020) in hepatic steatosis. Furthermore, previous results from our laboratory, which demonstrate that reduced 5-HT expression in the SI due to capsaicin-induced blockade of nerve signals is related to milder hepatic steatosis (Ko et al., 2021; Inoue et al., 2018; Kamimura et al., 2018; Nagoya et al., 2020), also support the conclusion that the 5-HT-liver-HTR2A axis is a key factor in NAFLD progression. These mechanisms were implicated in the modification of *Acaca*, *Fasn* and *Gpat1* genes, which are related to TG metabolism. In addition, similar effects were demonstrated upon administration of the HTR2A antagonist to HFD-fed WT and HFD-fed MC4RKO mice. Furthermore, compared to HFD-fed WT mice, HFD-fed MC4RKO mice showed more severe hepatic steatosis and TG content increase and milder recovery of these parameters after peripheral neural blockade and treatment with the HTR2A antagonist. These results indicate that diet-induced NAFLD is significantly related to the peripheral 5-HT-liver-HTR2A axis.

The effects of peripheral 5-HT in hepatic steatosis can be classified as those of either a direct hormonal signal to HTRs on the liver through portal circulation (Namkung et al., 2018; Li et al., 2018; Choi et al., 2018; Lam et al., 2010; Wang et al., 2020) or an indirect paracrine or autocrine factor that can modify the function of tight junctions and microbiota, as previously reported (Haub et al., 2011; Zhang et al., 2020). As a hormone, the function of 5-HT in a tissue is based on the type of the receptor that it activates. Here, we show that *Htr2a* (a gene involved in hepatic steatosis) and *Htr2b* (a gene activated in PH models) are needed for liver regeneration. Importantly, these results are supported by the expression of tissue-specific HTRs, such as HTR1B, HTR2A, HTR2B and HTR7, in the liver (Li et al., 2018), the relationship of HTR2A with hepatic steatosis (Li et al., 2018; Choi et al., 2018; Wang et al., 2020) and that of HTR2B with hepatocyte proliferation (Ruddell et al., 2008; Wouters et al., 2007). As the increase in 5-HT upregulates HTR2A and HTR2B expression in hepatocytes (Li et al., 2018), HTR activation is also expected to increase to mirror 5-HT increase in NAFLD and PH models. Nevertheless, further studies using *Tph1* KO mice are needed to understand whether HTR suppression is due to 5-HT decrease or autonomic nervous signal blockade. Next, as these changes were reproduced upon administration of a HTR2A antagonist, it emerges that the 5-HT-liver-HTR2A axis is activated by signals from the autonomic nervous system, which originate when the liver is exposed to diet-induced fatty changes. Most importantly, this pathway represents a potential therapeutic target. We also show that the serum levels of 5-HT were lower in NAFLD mice and NAFLD patients compared to controls, indicating that the 5-HT secreted from the SI binds to HTR2A, which reduces its serum concentration but promotes fatty acid synthesis. Moreover, a significant correlation between the liver CT scores (Park et al., 2011) and serum 5-HT concentration further supports the notion that serum concentrations of 5-HT can be a marker of NAFLD progression. Thus, based on these results, it is possible to attribute inconsistent 5-HT values in clinical NAFLD cases to the timing of the sample collection (Wang et al., 2020). Further studies are necessary to determine whether 5-HT can indeed be a biomarker for NAFLD progression, as it is taken up by platelets via the 5-HT transporter and stored in dense calcium-containing granules (Berger et al., 2009; Lesurtel et al., 2006).

We also examined the contribution of central 5-HT in the brain as it plays a role in appetite control as a neurotransmitter along with its receptor HTR2C via the MC4R signaling pathway (Namkung et al., 2015; Lam et al., 2010), independently of the 5-HT in the SI; however, no study has described the kinetics of central 5-HT secretion and its relationship with peripheral 5-HT in NAFLD. We showed that central 5-HT is higher in diet-induced NAFLD models, regardless of autonomic nerve signal blockade. In addition, as MC4RKO mice show enhanced appetites and obesity as well as increased expression of IGF-1 in the liver to decelerate steatohepatitis progression (Nagoya et al., 2020), IGF-1 might contribute to the milder increase in transaminase levels seen in MC4RKO mice than in other mouse models, in the early phase of the disease (Table S4). The protective effect of IGF-1 in liver injury has also been reported by other research groups (Nishizawa et al., 2012; Sobrevals et al., 2010). These results suggest that an increase in central 5-HT reduces appetite in these models to maintain homeostasis, independently of peripheral 5-HT. However, further experiments using mouse models with impaired central neural pathways, including NPY/AgRP and POMC, and employing a HTR2C agonist approved by the U.S. Food and Drug Administration for BW management (Bohula et al., 2018a,b) should be conducted to clarify the contribution of appetite control and the relationship between the central and peripheral autonomic neural pathways (Miller et al., 2021; Park et al., 2021; Nonogaki, 2022) in NAFLD pathology. Limitations of our study include the lack of real-time monitoring of physiological changes and activation of neurotransmitters in the brain and testing combinations of anatagonists of HTR2A and HTR2C. These experiments will provide further information regarding the peripheral and central nervous orchestration in NAFLD progression. In addition, the therapeutic effect of HTR antagonists should be tested in conditions in which NASH has progressed using mice that were fed with HFD for longer periods, for example, 16 weeks. Further studies are also necessary to understand cognate changes in platelet counts and other processes, although receptor-mediated uptake of 5-HT from portal circulation is one of the reasons for the observed decrease in serum concentration.

In conclusion, we show that autonomic nerve signal transduction from livers exposed to fatty changes activates 5-HT and that the 5-HT-liver-HTR2A axis is involved in diet-induced NAFLD pathogenesis (Fig. S1). The mechanisms in this pathology include enhanced 5-HT secretion into portal circulation, expression of HTR2A in the liver and activation of hepatic fatty acid synthesis-related gene expression. Moreover, the effect of central neural activity on NAFLD is separate from that of the peripheral autonomic nervous pathway. As neural blockade and HTR2A antagonists suppress this pathology, it is possible that 5-HT and its specific receptor-mediated signaling in a tissue represent novel therapeutic options for treating palatable food-induced NAFLD.

## MATERIALS AND METHODS

### Animals

All animal experiments were approved by and conducted in full compliance with the regulations of the institutional animal care and use committee, Niigata University, Niigata, Japan (SA00568). Male C57BL/6JJcl mice (*n*=65, 8-10 weeks old, 25-30 g, Charles River Japan, Yokohama, Kanagawa, Japan) and MC4RKO mice [*n*=50, 8-10 weeks old, 25-30 g, kindly provided by Dr Takayoshi Suganami (Nagoya University) and Dr. Yoshihiro Ogawa (Kyushu University)] were used. Animals were housed under standard conditions in specific pathogen-free facilities at a temperature of 20-23°C and humidity of 45-55%.

### Development of animal models

Mice were first divided into control, sham-operated (Sham) or capsaicin-treated (Cap) groups. Control mice were fed standard chow diet (SCD). Direct topical application of capsaicin (Wako Pure Chemical Industries, Osaka, Japan) dissolved in olive oil (50 mg/ml) was performed in the Cap group to deafferent the afferent sympathetic fibers from the liver. The celiac artery was exposed by laparotomy and wrapped in gauze immersed with (Cap) or without (Sham) capsaicin for 30 min, as previously described (Imai et al., 2008; Ko et al., 2021; Inoue et al., 2018; Nagoya et al., 2020). Although it has been reported that this method does not affect other nerves, including the vagus nerve (Hurr et al., 2019; Inoue et al., 2018; Warne et al., 2007), to verify that there was no effect on hunger or food intake due to Cap treatment, both groups were provided identical feed and their consumption was measured. These animals were further divided into nine groups as follows: mice fed a CDAA-containing 62% kcal fat, 18% kcal protein and 20% kcal carbohydrate (A06071302; Research Diets, New Brunswick, NJ, USA) for 4 or 8 weeks – Sham+CDAA (4W), Cap+CDAA (4W), Sham+CDAA (8W) and Cap+CDAA (8W); mice fed HFD containing 60% kcal fat, 20% kcal protein and 20% kcal carbohydrate (D12492; Research Diets) for 4 or 8 weeks – Sham+HFD (4W), Cap+HFD (4W), Sham+HFD (8W) and Cap+HFD (8W); MC4RKO mice fed SCD for 4 or 8 weeks – Sham+MC4RKO+SCD (4W), Cap+MC4RKO+SCD (4W), Sham+MC4RKO+SCD (8W) and Cap+MC4RKO+SCD (8W); MC4RKO mice fed HFD for 4 or 8 weeks – Sham+MC4RKO+HFD (4W), Cap+MC4RKO+HFD (4W), Sham+MC4RKO+HFD (8W) and Cap+MC4RKO+HFD (8W); and mice that underwent 70% hepatectomy (Sham+PH, Cap+PH) (Inoue et al., 2018; Nagoya et al., 2020). HFD was fed for 4 or 8 weeks in the present study according to previous reports (Crane et al., 2015; Choi et al., 2018) as well as our recent study (Ko et al., 2021) showing that *Tph1* expression and 5-HT synthesis were induced by feeding mice HFD for 4 or 8 weeks. The animal models are summarized in Table S1. Apart from these groups, mice fed SCD were used as controls (Fig. 1A). To assess the effect of sarpogrelate, a HTR2A antagonist, on normal and MC4RKO mice fed HFD for 4 weeks, sarpogrelate hydrochloride (SAR03A; Japanese Pharmacopoeia Reference Standard, Pharmaceutical and Medical Device Regulatory Science Society of Japan, Osaka, Japan) was administered in drinking water based on BW such that the animals received approximately 100 mg/kg sarpogrelate per day orally. To verify sarpogrelate consumption, platelet aggregation was assessed (Doggrell, 2004) as described previously using light transmission aggregometry (Born, 1962).

### Histological analysis

Tissue samples for immunohistochemical staining were collected from each group at appropriate time points. Tissues were collected 2 days after the procedure in PH groups, and at 4 or 8 weeks after the procedure in other groups. Samples were fixed in 10% formalin before paraffin embedding, five sections were obtained from each lobe of the liver and from the SI (10 μm) for each animal, and standard Hematoxylin and Eosin (H&E) staining and immunohistochemistry were performed. Hepatic adipose tissue was detected by H&E staining. Brain tissue was collected after perfusion of 4% paraformaldehyde, samples were fixed in 4% paraformaldehyde overnight at 4°C, embedded in paraffin and carefully sectioned to expose the requisite regions. The following antibodies were used for immunohistochemistry: mouse monoclonal anti-human 5-HT (M0758; Dako, CA, USA) at 1:200 dilution for the SI and 1:100 for the brain; mouse monoclonal anti-HTR2C (antiSR-2C, D-12) (sc-17797; Santa Cruz Biotechnology, TX, USA) at 1:100 dilution; mouse monoclonal anti-fos antibody (sc-166940; Santa Cruz Biotechnology) at 1:100 dilution with the Vectastain Elite ABC mouse IgG kit (PK-6102; Vector Laboratories, CA, USA) and DAB chromogen tablets (Muto Pure Chemicals, Tokyo, Japan); rabbit polyclonal anti-HTR2A antibody (GTX37799, GeneTex, LA, USA) at 1:100 dilution; rabbit polyclonal anti-HTR2B antibody (GTX32984, GeneTex) at 1:100 dilution; and rabbit polyclonal anti-NPY antibody (ab30914; Abcam, Cambridge, UK) at 1:500 dilution with the Vectastain Elite ABC rabbit IgG kit (PK-6101; Vector Laboratories); and DAB chromogen tablets (Muto Pure Chemicals). Images were captured for each tissue section randomly and quantitatively analyzed on ImageJ software (version 1.8.0_172; National Institutes of Health, Bethesda, MD, USA) with an RGB-based protocol, as reported previously (Vrekoussis et al., 2009).

### Quantitative analysis of hepatic lipids

Hepatic lipid content was analyzed by Skylight Biotech (Akita, Japan). Liver tissues were homogenized in chloroform/methanol (2:1, v/v), lipid extracts were prepared using the Folch method (Folch et al., 1957) and intrahepatic TC, TG and PL levels were measured using the enzymatic assay kits, Cholestest CHO, Cholestest TG and Pureauto S PL (Sekisui Medical, Tokyo, Japan), respectively.

### Quantitative real-time PCR

Total RNA was extracted from SI and liver tissue using the RNeasy Mini kit (QIAGEN, Hilden, Germany) and reverse transcribed into cDNA with the QuantiTect Reverse Transcription kit (QIAGEN). Gene expression was measured by quantitative real-time PCR using SYBR Green and the StepOnePlus System (Thermo Fisher Scientific, Waltham, MA, USA) and the results were analyzed using bundled software. Thermal cycling conditions were as follows: 95°C for 10 mins, followed by 40 cycles of 94°C for 15 s, 55°C for 30 s and 72°C for 30 s, and melting hold at 95°C for 15 s, 60°C for 1 min and 95°C for 15 s. Changes in gene expression were calculated using the 2−ΔΔCt method, with gene expression normalized to that of *Gapdh* for each sample. Primer sequences are listed in Table S2.

### Serum biochemical analysis

Blood samples were collected at 4 and 8 weeks, and AST, ALT, ALP, TC and TG levels were estimated in serum (Oriental Yeast, Shiga, Japan).

### Serum 5-HT analysis

Serum 5-HT levels were determined using a 5-HT ELISA kit (ADI-900-175; Enzo Life Sciences, NY, USA) at 1:24 dilution for mouse serum and at 1:16 dilution for human serum. The mouse serum was collected prior to tissue harvesting.

### Samples from NAFLD patients

The observational study protocol for clinical blood sample collection and CT images was approved by the Ethics Committee and Institutional Review Board of Niigata University School of Medicine (nos. 2192, G2015-0718, 2018-0092 and 2019-0023). Written informed consent was obtained from all patients for participation and data publication with complete anonymity. The study was conducted in accordance with the ethical guidelines of the Declaration of Helsinki, 1975. Stored serum samples from patients admitted to Niigata University Hospital between June 2014 to August 2020 and pathologically diagnosed with NAFLD by liver biopsy comprised the NAFLD group (*n*=31), whereas those from patients with no fatty infiltration on CT and any previous diseases constituted the control group (*n*=54). Clinical characteristics of the patients are shown in Table S3. Liver and spleen CT scores were calculated as reported previously (Zeb et al., 2012) and NAFLD activity score was used for pathological diagnosis (Kleiner et al., 2005). Selection criteria for patients with NAFLD were the absence of drinking history or regular drinking of <30 g per week for men and <20 g per week for women (Evidence-based Clinical Practice Guidelines for Nonalcoholic Fatty liver Disease/Nonalcoholic Steatohepatitis 2020, 2nd Edition; The Japanese Society of Gastroenterology and The Japan Society of Hepatology, 2020). Exclusion criteria were fatty liver secondary to viral hepatitis, autoimmune liver disease, drug-induced liver injury and genetic disorders (Chalasani et al., 2018).

### Statistical analyses

Data from each group are presented as the mean±s.d. and differences were evaluated by two-tailed unpaired Welch's *t*-test, two-way repeated-measure analysis of variance (ANOVA) or one-way ANOVA followed by Tukey's multiple comparison test, as applicable. For serum 5-HT levels in mice and patient samples, and for patient parameters, data are presented as the median and were compared using the Kruskal–Wallis test followed by Dunn's multiple comparison test or Mann–Whitney *U* test. Correlations were evaluated by performing Pearson's correlation test. All statistical analyses were computed using GraphPad Prism 8 software (version 8.4.2; MDF, Tokyo, Japan). Values of *P<*0.05 were defined as statistically significant.

## Supplementary Material

Supplementary information
