# Supplementary material for: Involvement of the liver-gut peripheral neural axis in nonalcoholic fatty liver disease pathologies via hepatic HTR2A
Source: Dis Model Mech. 2022 Jul 26;15(7):dmm049612. doi: 10.1242/dmm.049612 (PMC9346519; doi:10.1242/dmm.049612)
Supplement: Supplementary information [file dmm-15-049612-s1.pdf]

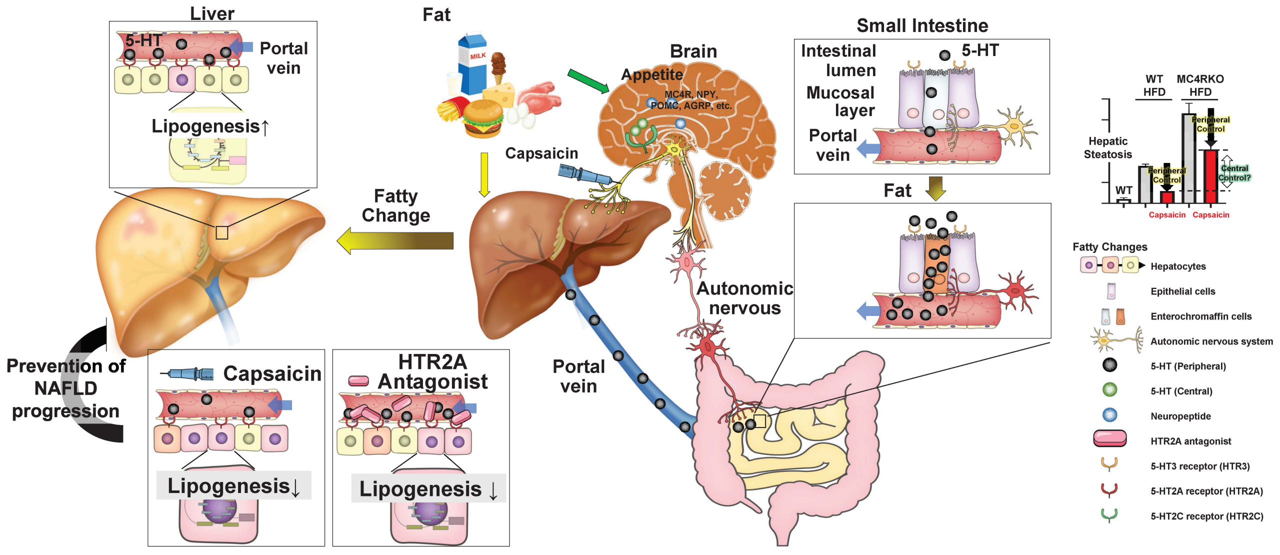

Fig. S1. Graphical abstract.

Table S1. Animal models

	Mice	Diet	Blockade of afferent sympathetic fibers	Follow up period
WT+SCD4W (Control)	C57BL/6JJcl	SCD	No	4 weeks
WT+SCD8W (Control)	C57BL/6JJcl	SCD	No	8 weeks
Sham+CDAA (4W)	C57BL/6JJcl	CDAA	No	4 weeks
Cap+CDAA (4W)	C57BL/6JJcl	CDAA	Yes	4 weeks
Sham+CDAA (8W)	C57BL/6JJcl	CDAA	No	8 weeks
Cap+CDAA (8W)	C57BL/6JJcl	CDAA	Yes	8 weeks
Sham+HFD (4W)	C57BL/6JJcl	HFD	No	4 weeks
Cap+HFD (4W)	C57BL/6JJcl	HFD	Yes	4 weeks
Sham+HFD (8W)	C57BL/6JJcl	HFD	No	8 weeks
Cap+HFD (8W)	C57BL/6JJcl	HFD	Yes	8 weeks
Sham+MC4RKO+SCD (4W)	MC4RKO	SCD	No	4 weeks
Cap+MC4RKO+SCD (4W)	MC4RKO	SCD	Yes	4 weeks
Sham+MC4RKO+SCD (8W)	MC4RKO	SCD	No	8 weeks
Cap+MC4RKO+SCD (8W)	MC4RKO	SCD	Yes	8 weeks
Sham+MC4RKO+HFD (4W)	MC4RKO	HFD	No	4 weeks
Cap+MC4RKO+HFD (4W)	MC4RKO	HFD	Yes	4 weeks
Sham+MC4RKO+HFD (8W)	MC4RKO	HFD	No	8 weeks
Cap+MC4RKO+HFD (8W)	MC4RKO	HFD	Yes	8 weeks
Sham+PH	C57BL/6JJcl	SCD	No	2 days
Cap+PH	C57BL/6JJcl	SCD	Yes	2 days

Table S2. The sequences of primers

**Gene name**	**Forward primer (5′ -3′)**	**Reverse primer (5′ -3′)**
Tph1	ACCATGATTGAAGACAACAAGGAG	TCAACTGTTCTCGGCTGATG
Htr2a	AAAGTTCTCTGCTGCGCTTC	CACGCAATGTTAATGCCATC
Htr2b	AAATAAGCCACCTCAACGCCT	TCCCGAAATGTCTTATTGAAGAG
Acaca	GCCTCTTCCTGACAAACGAG	TGACTGCCGAAACATCTCTG
Fasn	AAGCGGTCTGGAAAGCTGAA	AGGCTGGGTTGATACCTCCA
Gpat1	CCACAGAGCTGGGAAAGGTT	GTGCCTTGTGTGCGTTTCAT
Cpt1a	AGCTCGCACATTACAAGGACA	CCAGCACAAAGTTGCAGGAC
Mttp	TCTCACAGTACCCGTTCTT	TCTTCTCCGAGAGACATATCC
Pparg	GGTGTGATCTTAACTGCCGGA	GCCCAAACCTGATGGCATTG
Srebp1c	GGAGCCATGGATTGCACATT	GGCCCGGGAAGTCACTGT
Adgre1	TCAAGGACACGAGGTTGCTGA	CCAAGGGGCCAATCTGGAA
Ccl2	GGCTCAGCCAGATGCAGTTAA	AGCCTACTCATTGGGATCATCTT
Col1a1	ACATGTTCAGCTTTGTGGACC	TAGGCCATTGTGTATGCAGC
Tgfb1	GGGCTACCATGCCAACTTCTG	GAGGGCAAGGACCTTGCTGTA
Timp1	CCCTGCTCAGCAAAGAGC	TCACTCTCCAGTTTGCAAGG

Table S3. Clinical characteristics of the patients

Clinical characteristics	Control (n=54) Median (min–max)	NAFLD (n=31) Median (min–max)	*P*
Age	61 (30–75)	60 (18–76)	.7418
Male:Female	27:27	16:15	
Height (m)	1.61 (1.41–1.90)	1.62 (1.41–1.84)	.7046
BW (kg)	56.0 (34.2–111.0)	69.8 (48.2–140.0)	< .0001
BMI (kg/m^2)	22.1 (16.8–30.7)	25.6 (21.1–47.9)	< .0001
AST (U/L)	22 (14–57)	57 (26–166)	< .0001
ALT (U/L)	19 (8–62)	70 (16–267)	< .0001
ALP (U/L)	211 (109–337)	278 (107–510)	< .01
γ-GTP (U/L)	22 (7–159)	78 (19–729)	< .0001
T-Bil (mg/dL)	0.7 (0.4–2.2)	0.8 (0.4–2.9)	.0849
HbA1c (%)	5.6 (4.9–7.6)	6.1 (4.4–9.1)	< .0001
TG (mg/dL)	105 (29–456)	132 (31–464)	.0296
TC (mg/dL)	201 (146–280)	196 (124–282)	.1146
HDL-C (mg/dL)	63 (37–121)	50 (27–108)	.0100
LDL-C (mg/dL)	111 (63–256)	116 (33–177)	.8253
Plt (×10^4/μL)	23.4 (9.0–37.4)	17.7 (7.8–36.5)	.0066
FIB-4 Index	1.37 (0.45–3.82)	2.26 (0.36–10.47)	.0002
5-HT (ng/mL)	97.3 (27.1–281.4)	64.0 (9.2–254.5)	.0026
Liver CT score	62.61 (49.88–71.54)	40.42 (10.89–63.12)	< .0001
Spleen CT score	47.86 (38.51–59.76)	50.44 (28.84–57.40)	.7335
Liver-to-spleen CT score ratio	1.27 (1.05–1.59)	0.88 (0.22–1.35)	< .0001

BW, body weight; BMI, body mass index; AST, alanine aminotransferase; ALT, aspartate transaminase; ALP, alkaline phosphatase; γ-GTP, γ-glutamyl transferase; T-Bil, total bilirubin; HbA1c, hemoglobin A1c; TG, triglyceride; TC, total cholesterol; HDL-C, high-density lipoprotein cholesterol; LDL-C, low-density lipoprotein cholesterol; Plt, platelet; FIB-4, Fibrosis-*4*; 5-HT, 5-hydroxytryptamin. The values represent median (minimum–maximum), Mann–Whitney U test.

Table S4. Effect of autonomic neural signal transduction and HTR2A antagonist administration on the serum biochemical factors in mice

	Control	Sham+CDAA 4 W	Cap+CDAA 4 W	Sham+HFD 4 W	Cap+HFD 4 W	MC4RKO+ Sham+SCD 4 W	MC4RKO+ Cap+SCD 4 W	MC4RKO+ Sham+HFD 4 W	MC4RKO+ Cap+HFD 4 W	HFD 4 W, Sar (-)	HFD 4 W, Sar (+)
**AST (U/L)**	142.3 (41.6)	340.2 (109.9)	418.5 (17.4)	423.0 (85.9)	286.2 (99.4)	152.5 (39.9)	106.0 (11.0)	164.8 (41.3)	100.0 (19.7)	205.0 (16.7)	158.0 (7.6)
**ALT (U/L)**	32.3 (8.5)	99.0 (9.0)	189.0 (30.4) **	162.0 (25.6)	108.0 (22.0)	31.3 (3.4)	21.5 (1.7)	92.4 (17.4)	43.5 (11.1)	33.5 (4.9)	26.3 (1.7)
**ALP (U/L)**	131.9 (40.2)	400.5 (40.9)	607.5 (80.9)	477.0 (196.2)	400.5 (59.7)	243.3 (15.6)	217.6 (10.5)	144.9 (22.6)	118.2 (15.1)	218.0 (8.2)	192.0 (9.9)
**TC (mg/dL)**	60.9 (6.2)	67.5 (6.0)	76.5 (8.3)	139.5 (12.9)	85.5 (16.2) *	74.3 (4.9)	76.7 (1.4)	162.7 (5.6)	133.8 (5.8) *	138.5 (7.9)	146.0 (2.1)
**TG (mg/dL)**	50.1 (4.8)	58.5 (4.5)	72.0 (5.7)	103.5 (4.5)	63.0 (5.7) **	57.7 (7.8)	46.0 (9.9)	84.5 (18.4)	91.8 (12.5)	73.7 (12.4)	50.0 (8.4)

	Sham+CDAA 8 W	Cap+CDAA 8 W	Sham+HFD 8 W	Cap+HFD 8 W	MC4RKO+ Sham+SCD 8 W	MC4RKO+ Cap+SCD 8 W	MC4RKO+ Sham+HFD 8 W	MC4RKO+ Cap+HFD 8 W	MC4RKO+ HFD 4 W, Sar (-)	MC4RKO+ HFD 4 W, Sar (+)
**AST (U/L)**	334.8 (48.0)	459.0 (81.7) *	432.0 (100.5)	351.0 (41.8)	142.2 (28.9)	163.4 (30.1)	169.3 (39.2)	169.3 (36.8)	164.8 (41.3)	174.4 (23.9)
**ALT (U/L)**	166.5 (29.1)	175.5 (18.1)	139.5 (20.2)	193.5 (23.6)	29.3 (2.3)	43.3 (12.5)	141.5 (24.9)	59.3 (14.8) **	92.4 (17.4)	97.2 (18.0)
**ALP (U/L)**	661.5 (53.4)	643.5 (50.9)	496.8 (58.3)	626.4 (84.3)	188.7 (11.4)	178.6 (10.1)	189.5 (33.6)	149.5 (26.7)	216.8 (17.2)	241.3 (10.6)
**TC (mg/dL)**	108.0 (6.9)	81.0 (9.9)	58.5 (8.3)	148.5 (6.0) ***	73.5 (4.4)	76.0 (2.9)	180.1 (17.6)	155.3 (12.0)	153.0 (8.1)	144.0 (4.0)
**TG (mg/dL)**	58.5 (4.5)	67.5 (6.0)	117.0 (5.7)	90.0 (9.0)	47.3 (8.0)	76.4 (5.9) *	100.3 (10.1)	52.8 (7.4) ***	84.5 (18.4)	68.0 (11.7)

AST, alanine aminotransferase; ALT, aspartate transaminase; ALP, alkaline phosphatase; TC, total cholesterol; TG, triglyceride; Sar, sarpogrelate. The values represent mean (SD) (n = 6 for each group), Significance of the difference was evaluated using Welch's *t*-test. (**P* < .05, ***P* < .01, ****P* < .001 vs Sham group).
